# Mobile genetic elements explain size variation in the mitochondrial genomes of four closely-related *Armillaria* species

**DOI:** 10.1186/s12864-019-5732-z

**Published:** 2019-05-08

**Authors:** Anna I. Kolesnikova, Yuliya A. Putintseva, Evgeniy P. Simonov, Vladislav V. Biriukov, Natalya V. Oreshkova, Igor N. Pavlov, Vadim V. Sharov, Dmitry A. Kuzmin, James B. Anderson, Konstantin V. Krutovsky

**Affiliations:** 1 Laboratory of Forest Genomics, Genome Research and Education Center, Institute of Fundamental Biology and Biotechnology, Siberian Federal University, Krasnoyarsk, 660036 Russia; 2 Laboratory of Genomic Research and Biotechnology, Federal Research Center “Krasnoyarsk Science Center of the Siberian Branch of the Russian Academy of Sciences”, Krasnoyarsk, 660036 Russia; 3 Institute of Animal Systematics and Ecology, Siberian Branch of Russian Academy of Sciences, 630091 Novosibirsk, Russia; 4 Laboratory of Forest Genetics and Selection, V. N. Sukachev Institute of Forest, Siberian Branch of Russian Academy of Sciences, Krasnoyarsk, 660036 Russia; 5 Laboratory of Reforestation, Mycology and Plant Pathology, V. N. Sukachev Institute of Forest, Siberian Branch of Russian Academy of Sciences, Krasnoyarsk, 660036 Russia; 6 Department of High Performance Computing, Institute of Space and Information Technologies, Siberian Federal University, Krasnoyarsk, 660074 Russia; 7 Department of Biology, University of Toronto, Mississauga, ON l5L 1C6 Canada; 8 Department of Forest Genetics and Forest Tree Breeding, Georg-August University of Göttingen, 37077 Göttingen, Germany; 9 Laboratory of Population Genetics, N. I. Vavilov Institute of General Genetics, Russian Academy of Sciences, Moscow, 119333 Russia; 10 Department of Ecosystem Science and Management, Texas A&M University, College Station, TX 77843-2138 USA

**Keywords:** *Armillaria*, Duplications, Evolution, GIY-YIG, Homing endonucleases, Introns, LAGLIDADG, Mitochondrial genome, mtDNA, Mobile genetic elements

## Abstract

**Background:**

Species in the genus *Armillaria* (fungi, basidiomycota) are well-known as saprophytes and pathogens on plants. Many of them cause white-rot root disease in diverse woody plants worldwide. Mitochondrial genomes (mitogenomes) are widely used in evolutionary and population studies, but despite the importance and wide distribution of *Armillaria*, the complete mitogenomes have not previously been reported for this genus. Meanwhile, the well-supported phylogeny of *Armillaria* species provides an excellent framework in which to study variation in mitogenomes and how they have evolved over time.

**Results:**

Here we completely sequenced, assembled, and annotated the circular mitogenomes of four species: *A. borealis, A. gallica*, *A. sinapina,* and *A. solidipes* (116,443, 98,896, 103,563, and 122,167 bp, respectively). The variation in mitogenome size can be explained by variable numbers of mobile genetic elements, introns, and plasmid-related sequences. Most *Armillaria* introns contained open reading frames (ORFs) that are related to homing endonucleases of the LAGLIDADG and GIY-YIG families. Insertions of mobile elements were also evident as fragments of plasmid-related sequences in *Armillaria* mitogenomes. We also found several truncated gene duplications in all four mitogenomes.

**Conclusions:**

Our study showed that fungal mitogenomes have a high degree of variation in size, gene content, and genomic organization even among closely related species of *Armillara*. We suggest that mobile genetic elements invading introns and intergenic sequences in the *Armillaria* mitogenomes have played a significant role in shaping their genome structure. The mitogenome changes we describe here are consistent with widely accepted phylogenetic relationships among the four species.

**Electronic supplementary material:**

The online version of this article (10.1186/s12864-019-5732-z) contains supplementary material, which is available to authorized users.

## Background

The genus *Armillaria* consists of common saprophytic and pathogenic fungi that belong to the basidiomycete family *Physalacriaceae*. *Armillaria* parasitizes numerous tree species in forests of the Northern and Southern hemispheres. *Armillaria* species vary in virulence level and host spectrum and play important role in carbon cycling in forests [1, 2]. The life cycle of *Armillaria* is unique among basidiomycetes in that the vegetative phase is diploid, rather than dikaryotic [3]. Due to their capacity for vegetative growth and persistence through the production of rhizomoprhs, individuals of *Armillaria* are among the largest and oldest organisms on Earth [4–7].

Mitochondrial DNA (mtDNA) restriction maps of *A. solidipes* (formerly known as *A. ostoyae*) from different geographic regions were previously shown to differ greatly in size [8]. The interpretation was that biparental inheritance could increase cytoplasmic mixing and allow recombination in mitogenome. Although *Armillaria* mitogenome in natural populations is inherited uniparentally, the potential for transient cytoplasmic mixing, heteroplasmy, and recombination exists with each mating event [9]. Indeed the actual signature of recombination in the mitogenome of *A. gallica* has been detected [10]. No *Armillaria* mitogenomes, however, have been completely annotated and described previously. In this study, we report the complete sequences of the mitogenomes of *A. borealis*, *A. gallica*, *A. sinapina*, and *A. solidipes,* and describe their organization, gene content and a comparative analysis.

The main function of mitochondria is energy production via the oxidative phosphorylation. In addition to the primary function in respiratory metabolism and energy production, mitochondria are also involved in many other processes such as cell aging and apoptosis [11]. The limited number of genes in current mitogenomes can be likely explained by past transfer of many of their original genes into the eukaryotic nuclear genome, which occurred after a free-living ancestral bacterium was incorporated into an ancient cell as an endosymbiont [12–14]. According to the comparative mitogenome and proteome data, the organelle ancestor was likely related to *Alphaproteobacteria* [15–17]. In general, 14 conserved protein-coding genes involved in electron transport and respiratory chain complexes (*atp6*, *atp8*, *atp9, cob*, *cox1, cox2, cox3*, *nad1, nad2, nad3, nad4, nad4L* and *nad6*), one ribosomal protein gene (*rps3*), two genes encoding ribosomal RNA subunits - small (*rns*) and large (*rnl*) - and a set of tRNA genes have been found in fungal mitogenomes [18, 19]. Despite the relatively conserved gene content, however, fungal mitogenomes vary greatly in size: from 18,844 bp in *Hanseniaspora uvarum* [20] up to 235,849 bp in *Rhizoctonia solani* [21]. This wide size range might be explained in part by variation in length of intergenic regions, differences in number of introns (group I and II) and their various sizes [22]. For example, large mitogenome size of *Phlebia radiata* (156 Kbp) was explained by a large number of intronic and intergenic regions [23].

Mitogenomes may provide clues into the evolutionary biology and systematics of eukaryotes. Mitogenomes could be especially helpful to establish phylogenetic relationships when nuclear genes do not provide clear or substantial phylogenetic data to solve conflicting phylogenies [24]. Moreover, the high degree of polymorphism is found in some mitochondrial introns and intergenic regions making these DNA regions also useful in population studies [25, 26].

Most of the mitochondrial group I introns contain ORFs with GIY-YIG or LAGLIDADG homing endonucleases (HEGs) motifs [27–29]. HEGs represent one of the types of mobile genetic elements that are able to insert themselves into specific genome positions [30]. As shown, HEGs can expand mitogenome size, may cause genome rearrangements, gene duplications and import of exogenic nucleotide sequences through horizontal gene transfer (HGT) [31–34]. HEGs may be involved in the spread of group I introns between distant species [35, 36]. However, the scale, rate, and direction of intron transfer have not yet been sufficiently studied. According to one hypothesis, a common evolutionary trajectory is from an ancestor of high intron content to derivatives of low intron content via progressive loss [37–40], but further testing of this possibility is needed. More studies of intron losses and acquisitions in closely related lineages are required to shed light on their evolution.

The number of evolutionary and systematic studies based on comparative analysis of complete fungal mitogenome sequences has substantially increased recently [41–46], but the mitogenome of only one member (*Flammulina velutipes*) in the *Physalacriaceae* family (*Agaricales*, *Basidiomycota*) is now available [47]. Here, we describe the complete mitogenomes of four *Armillaria* species*.*

## Results

### Mitogenome organization

**Fig. 1:**
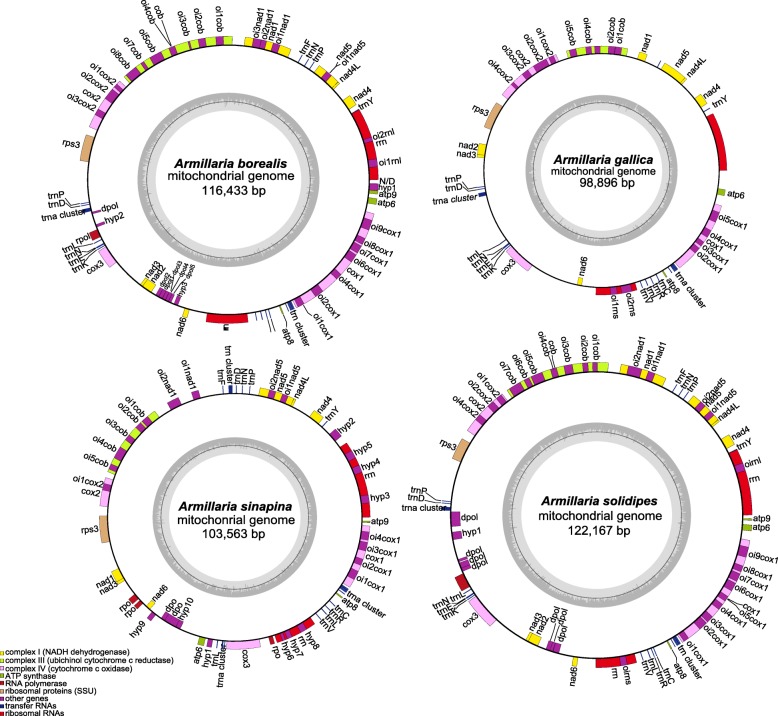
Circular complete graphic mitogenome maps of four *Armillaria* species: *A. borealis*, *A. solidipes*, *A. sinapina*, and *A. gallica*. Genes are transcribed in a clockwise direction. The inner gray rings show the GC content of these genomes

**Fig. 2:**
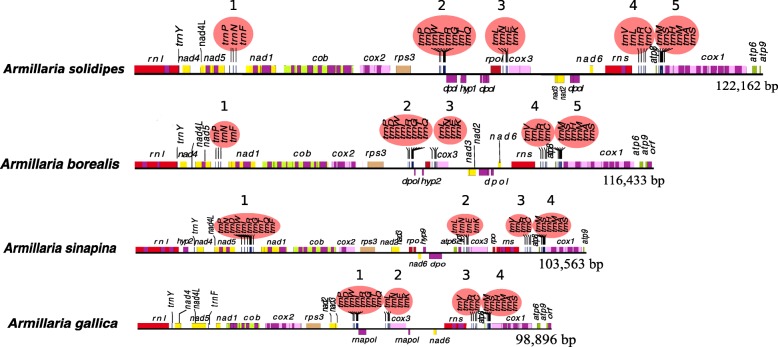
Linear complete graphic mitogenome gene maps of four *Armillaria* species: *A. borealis*, *A. solidipes*, *A. sinapina*, and *A. gallica* with tRNA gene locations highlighted by red ovals emphasizing clustering of some of them

**Fig. 3:**
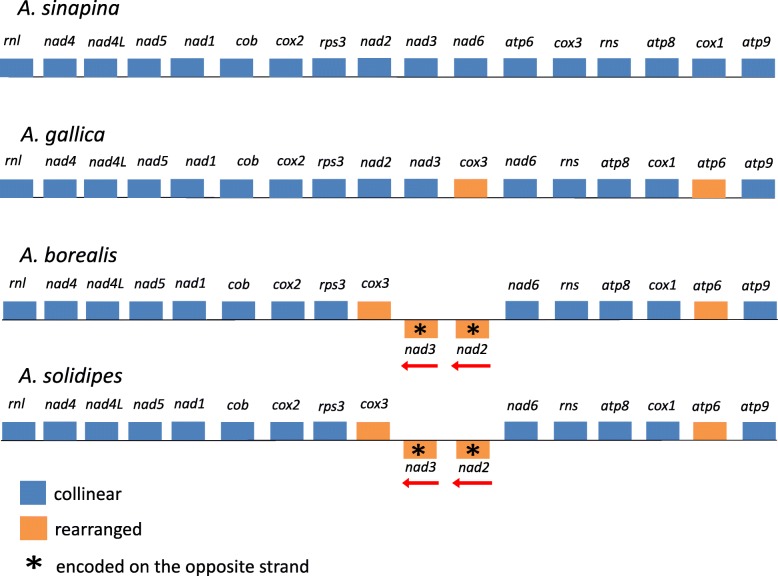
Gene order and rearrangements in mitogenomes of four *Armillaria* species: *A. borealis*, *A. solidipes*, *A. sinapina*, and *A. gallica*

The mitogenomes of *Armillaria* are 116,433 (*A. borealis*; GenBank accession number MH407470), 98,896 (*A. gallica*; MH878687), 103,563 (*A. sinapina*; MH282847), and 122,167 (*A. solidipes*; MH660713) bp circular DNAs (Fig. 1). The sequences were all AT-rich with similar AT content: 70.7% for *A. borealis*, 70.8% for both *A. gallica* and *A. solidipes*, and 71.5% for *A. sinapina.* We detected 16 tandem repeat or minisatellite loci in *A. borealis* and *A. sinapina*, 17 in *A. gallica*, and 11 in *A. solidipes* (Additional file 1: Table S1) using Tandem Repeats Finder (https://tandem.bu.edu/trf/trf.html). The length of the longest tandem motif was 41 bp in *A. borealis*, 27 bp in *A. gallica*, 23 bp in *A. sinapina*, and 37 bp in *A. solidipes* with two repeats in each species. In general, most tandem repeat loci contained two or three repeats. In addition, we also searched for microsatellite or simple sequence repeat (SSR) loci using SciRoKo (https://kofler.or.at/bioinformatics/SciRoKo) and found 8 SSR loci in *A. borealis*, 12 in *A. gallica*, 15 in *A. sinapina*, and 10 in *A. solidipes* (Additional file 2: Table S2). The comparisons of the whole mitogenomes using MAUVE identified conserved genomic blocks, as well as sequences rearrangements in several locations (Figs. 2 and 3).

Each mitogenome contained 15 protein-coding genes: three ATP-synthase complex F0 subunit genes (*atp6, atp8*, and *atp9*), three complex IV subunits genes (*cox1*, *cox2*, and *cox3*), one complex III subunit gene (*cob*), seven electron transport complex I subunits genes (*nad1*, *nad2*, *nad3*, *nad4*, *nad4L*, *nad5*, and *nad6*), one ribosomal protein gene (*rps3*), as well as large and small ribosomal subunits RNA genes (*rnl*, and *rns*) that are encoded on both strands. In all four mitogenomes the *nad2* and *nad3* and *nad4L* and *nad5* genes were linked with a slight overlap: the stop-codon of *nad2* overlapped the following start codon of *nad3* by one nucleotide, and the stop codon of *nad4L* also overlapped the following start codon of *nad5* by one nucleotide*.* All of these protein-coding genes are encoded on the same DNA strand, except for *nad2* and *nad3* that start with the typical translation initiation codon ATG, but are encoded on the opposite strand in *A. borealis* and *A. solidipes* (Fig. 3).

Some exons in protein-coding genes were difficult to annotate using MFannot due to their particularly small size. The smallest exons were found in the *cob*, *cox1* and *cox2* genes, such as 15 bp long 10th exon in *cox1* and 12 bp long exon 6 in *cob* in *A. borealis*, 12 bp long exon 5 in *cob* in *A. sinapina*, 15 bp long exon 9 in *cox1* and 15 bp long exon 3 in *cox2* in *A. solidipes*. Therefore, these exons were annotated manually.

In total, 26, 24, 25, and 26 tRNA genes were annotated in the mitogenomes of *A. borealis*, *A. gallica*, *A*. *sinapina*, and *A. solidipes,* respectively. Similar to most fungal mitogenomes studied so far, the tRNA genes in all four mitogenomes were mainly clustered (Fig. 2), except the tRNA-Tyr gene (*trnY*), which was located between *rnl* and *nad4* in all four *Armillaria* mitogenomes, and the tRNA-Phe gene (*trnF*) that was located along outside of clusters in all mitogenomes except *A*. *sinapina*. *A. borealis* and *A. solidipes* had the same five clusters. *A. gallica* and *A. sinapina* had four similar clusters that were only slightly different from five clusters in *A. borealis* and *A. solidipes.* The clusters were only slightly different in composition and location. All different tRNA genes were presented by a single copy except the tRNA-Pro gene (*trnP*) that had two copies in *A. borealis* and *A. solidipes.*

### Gene order

The whole-genome alignments of the mitogenomes of *A. borealis*, *A. gallica*, *A. sinapina*, and *A. solidipes* revealed a predominant pattern of conservation of gene order and orientation, but with distinct variations (Figs. 2 and 3). *A. borealis* and *A. solidipes* had the same gene order and orientation, while *A. gallica* and *A. sinapina* contained gene rearrangements between *nad3* and *atp9* genes. *A. gallica* is different from *A. borealis* and *A. solidipes* only by a single inversion having the *nad2-nad3-cox3* gene order vs. *cox3-nad3-nad2*. In addition, *nad3* and *nad2* are translated in the opposite direction from the opposite strand in *A. borealis* and *A. solidipes*. In *A. sinapina* the *cox3* and *atp6* genes were transposed and rearranged. The rearrangements are consistent with *A. borealis* and *A. solidipes* being sister species and *A. sinapina* and *A. gallica* being more distantly related [48, 49].

### Codon usage

The codon usage frequencies for 14 protein-coding mitochondrial genes were determined for each *Armillaria* species (Additional file 3: Table S3). The start codon ATG was detected across all four species in all genes ended with the TAA stop codon except *atp9* gene, which ended with TAG. The AT-rich codons were predominant, and the most-frequently used codons were invariant: TTA (Leu,10.77–11.03%), TTT (Phe, 5.63–5.92%), ATA (Ile, 5.18–5.28%), ATT (Ile 5.14–5.30%), GGT (Gly 3.09–3.19%). On the other hand, the СGC (Arg) codon was universally absent in all four species. Moreover, several codons were under-represented (having frequency < 0.5%), such as TGC (Cys, 0.02%), AGG (Arg, 0.02–0.05%), CGG (Arg, 0.10–0.14%), CGA (Arg, 0.17%), CGT (Arg, 0.05–0.07%), AGC (Ser, 0.17–0.19%), TGG (Trp, 0.29–0.36%), CAG (Gln, 0.24–0.26%), and CCC (Pro, 0.43–0.50%). Similar to other fungal studies, mitochondrial genes of *Armillaria* had a high number of AT-rich codons, and similar codon frequencies are found in other fungal mitogenomes [22].

### Introns and plasmid-related sequences

In total, 26 introns were found in seven out of 15 protein-coding genes in *A. borealis*, 27 introns in six genes in *A. solidipes*, and 18 introns in six genes in *A. sinapina* and *A. gallica* (Table 1).

**Table 1:** Number of introns in seven protein-coding genes in mitogenomes of four *Armillaria* species

Species	*cox1*	*cox2*	*cox3*	*cob*	*nad1*	*nad5*	*atp9*	Total
*A. borealis*	9	4	1	8	2	1	1	26
*A. solidipes*	9	5	2	7	2	2	–	27
*A. sinapina*	4	2	2	6	2	2	–	18
*A. gallica*	5	5	2	4	–	1	1	18

The size of the introns ranged from 189 bp (intron in *atp9* in *A. gallica*) to 2615 bp (intron 2 in *nad1* in *A. solidipes*). The average length of introns in all four species was 1902 bp. All introns were classified into group I, and some of them were further classified into subgroups IA (1), IB (10), and I-derived (7) in *A. borealis*, IB (10) and I-derived (6) in *A. gallica*, IB (5), ID (1), and I-derived (5) in *A. sinapina*, and IB (10) and I-derived (8) in *A. solidipes* (Additional file 4: Table S4).

**Fig. 4:**
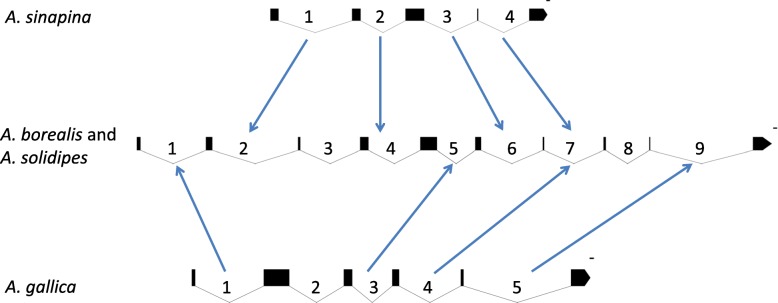
Introns (1–9) of the *cox1* gene in four *Armillaria* species: *A. borealis*, *A. solidipes*, *A. sinapina*, and *A. gallica*. Black boxes represent exons. Arrows depict homologous or orthologous introns

Some introns in the same genes demonstrated only partial identity or orthology. For example, intron 2 in *cox1* had 100% sequence similarity and the same insertion point in *A. borealis* and *A*. *solidipes*, but it showed no sequence similarity with intron 2 in *cox1* of *A. gallica*. Intron 5 in *cox1* had the same insertion point in *A. borealis and A. solidipes*, but had different insertion point in *A. gallica* and was completely identical (with 100% sequence similarity) to intron 3 in this species, but was not found in *A. sinapina*. However, all introns in *cox1* of *A. sinapina* seemed orthologous to those in *A. borealis* and *A. solidipes*. In total, nine orthologous introns could be identified for *cox1* between *A. borealis* and *A. solidipes*, four such introns among *A. borealis, A. solidipes* and *A. sinapina,* four introns among *A. borealis, A. solidipes* and *A. gallica*, and only one orthologous intron between *A. sinapina* and *A. gallica* (Fig. 4). Therefore, due to the presence and absence of various introns, the size of the *cox1* gene varied from 8132 bp in *A. sinapina* to 15,987 bp in *A. borealis*. Here again, the pattern of change is consistent with *A. borealis* and *A. solidipes* as sister species and *A. gallica* and *A. sinapina* as more distantly related.

Overall, *A. borealis* shared 25, 15 and 15 homologous or orthologous introns with *A. solidipes*, *A. sinapina* and *A. gallica*, respectively; *A. solidipes* 25, 15 and 16 with *A. borealis*, *A. sinapina* and *A. gallica*, respectively; *A. sinapina* 15, 15 and 9 with *A. borealis*, *A. solidipes* and *A. gallica*, respectively. *A. gallica* 16, 15 and 9 introns with *A. solidipes*, *A. borealis* and *A. sinapina*, respectively. The unique introns from each mitogenome were blasted against the NCBI GenBank database and revealed some similar sequences even in distantly related fungal mitogenomes (Table 2). In total, 11 unique introns were found in the four species: three in *A. borealis* (introns 1 and 6 in *cob* and intron 2 in *cox2* that were 2288, 551 and 2585 bp long, respectively); five in *A. solidipes* (intron 1 in *nad5*, intron 3 in *cob*, introns 2 and 3 in *cox2*, and intron 1 in *cox3* that were 1199, 1560, 1567, 381 and 1668 bp long, respectively). *A. sinapina* contained one unique intron 2 in *nad1* (2547 bp), and *A. gallica* contained one unique intron 2 in *cox1* (1320 bp).

**Table 2:** The unique introns based on the BLAST analysis

Gene	Intron	Position	BLAST hits						
			Identities	Cover	Species	Division	Accession	Gene	Intron
*A. borealis*									
*cob*	1	1094..1917	663/884 (75%)	41%	*Lentinula edodes*	*Basidiomycota*	AB697988.1	*cob*	1
*cob*	6	no significant hits							
*cox2*	2								
*A. solidipes*									
*nad5*	1	679..1093	294/432 (68%)	34%	*Leptogium hirsutum*	*Ascomycota*	KY457237.1	*nad5*	2
*cob*	3	507..962	313/467 (67%)	30%	*Ganoderma sinense*	*Basidiomycota*	KF673550.1	*cob*	3
*cox2*	2	339..848	345/518 (67%)	32%	*Rhizoctonia solani*	*Basidiomycota*	KC352446.1	*cox2*	2
*cox2*	3	no significant hits							
*cox3*	1								
*A. gallica*									
*cox1*	2	no significant hits							
*A. sinapina*									
*nad1*	2	no significant hits							

Many introns contained ORFs encoding proteins which have similarities with homing endonucleases of LAGLIDADG (12 ORFs) and GIY-YIG (7 ORFs) families in *A. sinapina*, 15 and 9 in *A. borealis*, 17 and 8 in *A. solidipes*, 13 and 4 in *A. gallica* (Table 3). Among free-standing ORFs, we found two possible homing endonuclease genes in *A. sinapina*, the first was located between *rnl* and *nad4* (LAGLIDADG) and the second was between *atp6* and *cox3* (GIY-YIG)*.* One possible free-standing homing endonuclease was found in each *A. borealis* and *A. gallica* (LAGLIDADG) next to *atp9*.

**Table 3:** Number of ORFs representing homing endonucleases of LAGLIDADG and GIY-YIG families in introns of seven genes in mitogenomes of four *Armillaria* species

Gene	LAGLIDADG				GIY-YIG			
	*A. sinapina*	*A. borealis*	*A. solisipes*	*A. gallica*	*A. sinapina*	*A. borealis*	*A. solidipes*	*A. gallica*
*rnl*	2	1	1	1	1	1	1	1
*cox1*	1	5	5	2	3	4	4	2
*cox2*	1	2	3	3	0	0	0	1
*cob*	4	5	5	5	1	1	1	0
*nad1*	0	0	0	0	2	3	2	0
*nad5*	2	1	2	0	0	0	0	0
*rns*	2	1	1	2	0	0	0	0
Total	12	15	17	13	7	9	8	4

We found ORFs in all four species that had homology with another type of mobile genetic elements – plasmid-like elements: five ORFs in *A. sinapina*, eight in *A. borealis*, six in *A. solidipes*, and two in *A. gallica*. In *A. borealis* and *A. solidipes* three plasmid ORFs were located between *rps3* and *cox3*, two of them were similar to the DNA polymerase and RNA polymerase genes, and one ORF had unknown function. These ORFs were not present in mitogenomes of *A. gallica* and *A. sinapina*. Regions located between *rps3* and *cox3* in the mitogenomes of *A. borealis* and *A. solidipes* contained also ORFs that encode a 2034 bp (*in A. solidipes*) and 2646 bp (in *A. borealis*) long fragment of the DNA polymerase gene and a nearby located 1053 bp (in *A. solidipes*) and 1080 bp (in *A. borealis*) long fragments of the RNA polymerase gene. They were not present in the *A. sinapina* mitogenome.

In *A. gallica*, two plasmid-related ORFs (1173 and 681 bp) were located between *nad3* and *cox3* and one (375 bp) between *cox3* and *nad6*. All of them were similar to the RNA polymerase genes.

In *A. sinapina*, two plasmid-related ORFs were located between *nad3* and *nad6* and represented 774 and 549 bp long RNA-polymerase genes. In addition, four ORFs were located between *nad6* and *atp6* and represented two 606 and 609 bp long genes that may encode hypothetical proteins with unknown function and other two 534 and 1707 bp long genes that were similar to the DNA-polymerase genes and arranged one after another.

### Gene duplications

The mitogenomes of *A. solidipes* and *A. sinapina* contained a common region with homology to *atp9* and located on a complementary strand in the *rnl* gene. It consisted of an 89 bp long sequence of the *atp9* gene with 87% identity with the 89 bp long fragment of the 222 bp long original gene in both species. Although *A. borealis* and *A. gallica* lacked copies in these regions, they contained 47 bp and 54 bp long copies of the exon 2 of the *atp9* gene, respectively, which were located upstream to the *atp9* 222 bp long coding sequence, next to the LAGLIDADG free-standing ORF.

### Mitogenome size variation

**Fig. 5:**
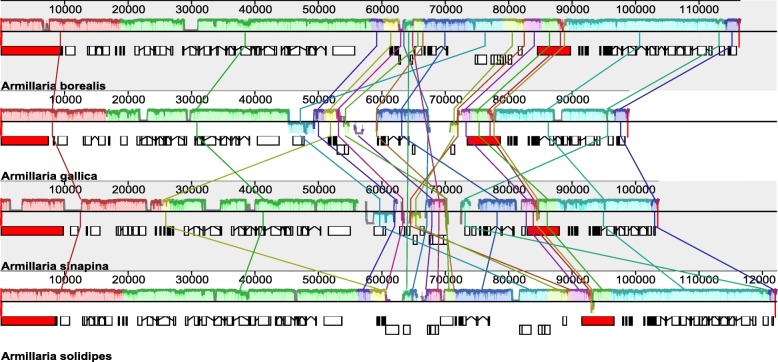
Structural alignments of *Armillaria* mitochondrial genomes based on the Mauve analysis. The color blocks indicate the homologous regions between the sequences shared by all mitogenomes. Gaps show unique sequences for each mitogenome. Blocks drawn under the horizontal line represent inverted sequence orientations

The mitogenomes described in this study showed substantial size variation, with *A. solidipes* having the largest (122,167 bp) and *A. gallica* the smallest (98,896 bp) mitogenomes. Different numbers and sizse of introns and intergenic regions are the simplest explanation for this variation. The mitogenomes with 27 introns in *A. solidipes* and 26 in *A. borealis* were larger than mitogenomes in *A. sinapina* and *A. gallica* with only 18 introns. The largest gene in *A. borealis, A. solidipes* and *A. gallica* was *cox1* that contained 9, 9 and 5 introns, respectively, contributing to its large size (15,955, 15,986 and 9624 bp, respectively). In *A. sinapina*, the largest gene was *cob*, which had 6 introns and was 9649 bp. The longest intron (2615 bp) was observed in the *A. solidipes* mitogenome (intron 2 of the *nad1* gene), and the shortest intron was 189 bp long in the atp*9* gene of the *A. gallica* mitogenome. Exons of the protein-coding genes and sequences of the rRNA genes covered 29% (29,159 bp) of mitogenome in *A. gallica*, 30% (31,139 bp) in *A. sinapina*, 26% (30,781 bp) in *A. borealis* and 24% (29,241 bp) in *A. solidipes*. The total length (and percentage) of intergenic sequences together with all introns and intergenic ORFs was 69,737 (71%), 72,424 (70%), 85,652 (74%) and 92,921 (76%) bp in *A. gallica*, *A. sinapina*, *A*. *borealis* and *A. solidipes*, respectively. These estimates were confirmed by the whole mitogenome comparative alignments generated by MAUVE, which showed variation in the intronic and intergenic regions (Fig. 5).

### Mapping RNA-seq reads to mitogenomes

The annotation of conserved protein-coding genes and rRNA genes was validated by mapping RNA-seq reads to mitogenomes. After filtering, 2,371,666 and 1,844,578 high quality reads of *A. borealis* and *A. sinapina*, respectively, were retained for mapping to their mitogenomes. In total, 258,471 reads were mapped to the *A. borealis* mitogenome and 227,565 reads to the *A. sinapina* mitogenome. In *A. borealis* and *A. sinapina* 17 and 16 genes were covered by the RNA reads, respectively (Additional file 5: Table S5). Much less number of reads were mapped to the *A. gallica* and *A. solidipes* mitogenomes using RNA-seq data for these two species downloaded from NCBI Sequence Read Archive (SRA). Only 11 genes were covered by 566 reads in the *A. gallica* mitogenome and eight genes by 442 reads in the *A. solidipes* mitogenome. Low mapping coverage data for these two species can be likely explained by a very low quality of transcriptome reads for *A. gallica* and *A. solidipes*, long stretches of which contained anonymous nucleotides (N). When *A. borealis* RNA reads were used for mapping to the *A. gallica* and *A. solidipes* mitogenomes, 16 genes were covered in both species by 251,265 and 255,744 reads, respectively.

### Phylogeny

**Fig. 6:**
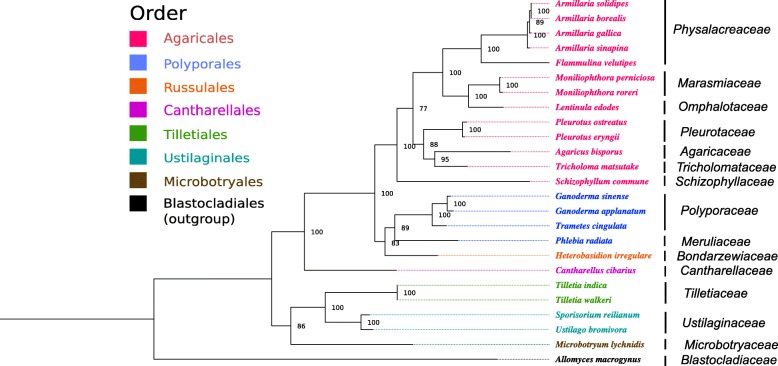
Maximum Likelihood phylogenetic tree based on protein sequences representing totally 3645 amino acids of 14 concatenated protein-coding mitochondrial genes from 25 fungal taxa. The numbers next to the cluster nodes represents bootstrap values based on 10,000 replicates

Phylogenetic analyses were performed using protein sequences consisting of totally 3645 amino acids of 14 concatenated protein-coding mitochondrial genes representing 25 fungal taxa. The maximum likelihood (ML) phylogenetic tree (Fig. 6) demonstrated that *Agaricales* formed a monophyletic group with a strong bootstrap support (100%). Within this clade, four families, *Physalacriaceae*, *Marasmiaceae*, *Omphalotaceae* and *Pleurotaceae* can be recognized as very strongly supported subclades also with 100% bootstrap support. *Physalacriaceae* that includes *Armillaria* species together with *Flammulina velutipes* was a sister lineage to *Marasmiaceae*. *A. solidipes* grouped with *A. borealis,* while *A. sinapina* and *A. gallica* seemed to be more distantly related. The protein changes are consistent with the established phylogenetic relationships of the four species [48, 49].

## Discussion

*A. borealis*, *A. solidipes*, *A. sinapina* and *A. gallica* are closely related, but their mitogenomes vary substantially in size. Some fungal mitogenomes contain multiple repeat sequences that represent mobile elements capable of inflating genome size, as has been observed, for example, in the mitogenome of *Ophiocordyceps sinensis* [44] and *Rhizoctonia solani* [21]. However, sequence repeats were not found in all four *Armillaria* mitogenomes, reflecting, that accumulation of repeats is not the reason for size variation between *Armillaria* species.

Although gene duplication could not explain genome size variation, we found that the mitogenomes of *Armillaria* were rich in mobile genetic elements that could be involved in increasing genome size. In earlier publications (see [50] for review) it was suggested that fungal mitogenome size variation could result, at least in part, from variation in the number and size of introns and in the length of intergenic sequences. Our data on variation of mitogenome sizes are consistent with this suggestion and with published studies that attributed expansions of fungal mitogenomes to intron sequences [24, 26, 37, 51], as well as plasmid-related sequences [32, 52]. The extent to which the intron sizes and numbers can affect mitogenome size is evident from the *cox1* gene in *A. borealis and A. solidipes*, which was the largest reservoir of 9 introns. This gene in these two species was more than double the size of this gene in *A. sinapina* due to additional introns. This observation was not limited to the *cox1* gene, but was true also for all other mitochondrial genes harboring introns. Long stretches of intergenic sequences also seemed to affect the mitogenome sizes of these species. In other fungi, these regions also harbor ORFs that have been associated with mitogenome size variation [29, 31, 37].

Introns in the four *Armillaria* mitogenomes confirmed the diversity of mitochondrial introns within the same genes reported in other published studies [53, 54]. Intron content of the *Armillaria* mitochondrial genes was notably diverse within the *cox1*, *cob*, *nad1*, *nad2* and *nad5* genes. Fungal mitochondrial introns showed a wide range of diversity even among closely-related species from the same genus [55, 56]. Thus, the diversity of introns in the mitogenomes of *Armillaria* is a common fungal feature, which can also explain mitogenome size variations.

In fungal mitogenomes, intron acquisition can occur through vertical and horizontal transmissions, and insertions do not necessarily occur at homologous gene positions [38, 54]. A high level of sequence similarity was observed between the introns of the *A. borealis* and *A. solidipes* mitogenomes, most of which encoded similar HEGs and had similar insertion points. Introns that lacked sequence similarities may be examples of independent evolutionary histories implying multiple acquisitions [24, 27]. For the few introns identified in these mitogenomes that did not share sequence similarities, similar sequences could be identified from other fungal species. This suggests that these introns were probably acquired by horizontal transfer. Our study confirmed published data that some fungal mitochondrial introns can contain ORFs that encode HEGs of the LAGLIDADG or GIY-YIG families [57]. These ORFs did not have the same start codons that are common in other fungal mitochondrial genes. Similarly to Stone et al. [24] we also observed in *A. sinapina*, *A. borealis* and *A. gallica* that these ORFs were free standing in mitogenomic regions between typical oxidative phosphorylation genes*.*

We also found that *Armillaria* mitogenomes contained rearrangements. Based on the genome-wide alignment, we surmise that mitogenome rearrangements are related to the presence of non-conserved plasmid-related sequences and homing endonucleases ORFs, which are common within intergenic regions of fungi [28, 58]. Most of these sequences represented truncated sequences of DNA polymerase and RNA polymerase genes, while some ORFs had unknown functions in *Armillaria*. In addition to the introns, plasmids represent mobile genetic elements often found in the mitochondria of fungi and plants and contain two ORFs, one of which encodes a family B DNA polymerase, and the other of which encodes the RNA polymerase subunit [59]. These genetic element insertions have been shown to be associated with or promote genomic rearrangements through non-homologous recombination [60, 61]. Plasmid-related DNA polymerase genes were found in mobile mitochondrial plasmids that occured either as free linear or circular DNAs, and have been shown to also insert into mitogenomes [30, 62, 63]. In the comparison of four closely related *Armillaria* species the presence of plasmid-related sequence insertions in the *rps3-atp9* region was observed. In *A. sinapina* and *A. gallica* they were sufficiently divergent from each other and from those in *A. borealis* and *A. solidipes*, which had more similarity between plasmid-related sequences in their mitogenomes, and consequently were most likely resulted from plasmid insertion events independent to those in *A. sinapina* and *A. gallica*. The sequences of the two DNA polymerase and RNA polymerase genes in *A. borealis* and *A. solidipes* had a high similarity (99% nucleotide identity), suggesting the common origin of these insertions. Other plasmid-related ORFs in the *A. gallica* and *A. sinapina* mitogenomes had low sequence similarity (< 45%) with each other and with *A. borealis* and *A. solidipes* indicating that these genes could have diverse origins. Therefore, acquisition of these genes in *A. sinapina* and *A. gallica* seems to have independent evolutionary origins which were then followed by the accumulation of mutations.

Mitogenomes of *Armillaria* species contained truncated copies of some genes, such as *atp9*. The high nucleotide identity (90–95%) between copies in all three genomes was accompanied by the high level of synteny around the duplicated gene regions. In *A. borealis* and *A. gallica* the copy was located nearby original *atp9* gene, but in *A. solidipes* and *A. sinapina* it was on the minus strand within *rnl* gene. In *A. borealis* and *A. gallica* the second exon of *atp9* was duplicated, while *atp9* in *A. sinapina* and *A. solidipes* did not have an intron, but had a truncated copy that corresponded to the first exon of *atp9* in *A. borealis* and *A. sinapina*. Duplications and copies of *atp9* were found also in mitogenomes of other fungal species, which implies that gene duplications (often accompanied or followed by their truncation) is a frequent process in fungal mitogenomes [24, 41].

According to the ML phylogenetic tree *A. solidipes* was very closely related to *A. borealis,* apart from *A. sinapina* and *A. gallica* that were less related to each other, which is consistent with previous phylogenetic studies based on a few genetic markers such as ITS*, tef1-α* and *β-tubulin* [64] or on analysis of six gene regions such as 28S, EF1α, RPB2, TUB, gpd and actin-1 [48]. Moreover, it is important to point out that there was an agreement between the phylogenetic grouping and the mitogenome organization of these species considering the fact that *A. borealis* and *A. solidipes* had the same gene order while *A. sinapina* and *A. gallica* each had the different unique gene order.

What is the impact of the mitogenome changes observed here on phenotype and fitness? The simplest hypothesis is that the mitogenome changes are neutral with respect to fitness and that their random accumulation parallels the species phylogeny [48, 49]. There may be an experimental test of this hypothesis. If mitogenome variation exists within species, then mitogenome recombinants could be obtained in laboratory matings [65]. Mitogenomes variants with precisely the same nuclear genome could then be tested for differences in phenotype and/or fitness traits in the laboratory.

## Conclusions

The mitogenomes of *A. borealis*, *A. solidipes, A. sinapina* and *A. gallica* had similar gene content. They contained 14 protein-coding conserved genes involved in oxidative phosphorylation and electron transport. The *rnl, rns, rps3* and tRNA genes were also found in all four mitogenomes. The genes order was the same in *A. borealis* and *A. solidipes*, but different in *A. sinapina* and *A. gallica*, consistent with the widely accepted interpretation of species phylogeny. Comparative analyses showed high size variation of these mitogenomes, which appeared due to the different number and size of introns and intergenic regions. Several introns seemed to have been acquired independently through intron-encoded homing endonucleases ORFs mobility. The frequent lack of sequence identity between introns identified in this study but high sequence identity with the sequences of other fungi available in the NCBI GenBank suggests their possible acquisition via horizontal transfer between even distantly related fungal species. However, a further comparative evolutionary analysis is required for these genes. The studied mitogenomes provide useful resources for these and other comparative studies.

## Methods

### DNA isolation

Mitogenomes were assembled from DNA sequences obtained using the total genomic DNA isolated from fungal mycelium without prior mtDNA isolation or enrichment. *A. borealis* and *A. sinapina* mycelia were collected in Western Siberia from *Abies sibirica* trees. The mycelia were fixed and stored for 2 days at 4 °C in the RNA stabilization solution RNA*later* (Thermo Fisher Scientific Company, Waltham, Massachusetts, USA). The RNA*later*-fixed mycelium was then quickly ground in acid-washed and autoclaved mortar. DNA was isolated using a modified version of the hot-CTAB extraction at 65 °C [66] followed by chloroform double-wash. Total DNA was precipitated within one hour with isopropanol at 4 °C, centrifuged at 6500 g for 30 min at 4 °C, washed twice with 70% ethanol, and eluted with 50 μl RNase-free water. Integrity and amount of the isolated total DNA were examined by 1.5% (wt/vol) agarose gel electrophoresis and using the NanoDrop 1000 Spectrophotometer (Thermo Fisher Scientific Company, Waltham, Massachusetts, USA). DNA was quantified on the Qubit 2.0 Fluorimeter (Thermo Fisher Scientific Company, Waltham, Massachusetts, USA).

### DNA sequencing and de novo assembly

The total DNA isolated from *A. borealis* and *A. sinapina* was sequenced in the Laboratory of Forest Genomics (Genome Research and Education Center, Siberian Federal University, Krasnoyarsk, Russia) using the Illumina MiSeq platform. The pair-end (PE) libraries with a mean insert size of 250 bp were subjected to 2 × 250 cycles of PE sequencing. Adapter sequences were trimmed and short reads were filtered using Trimmomatic v. 0.36 [67] with minimum quality of 19 and minimum length of 35 bp. Quality was assessed using FASTQC v. 0.11.5 (http://www.bioinformatics.babraham.ac.uk/projects/fastqc). The obtained sequence reads were assembled into contigs and scaffolds using the CLC Assembly Cell v. 5.0.0 (QIAGEN Bioinformatics, Hilden, Germany; https://www.qiagenbioinformatics.com/products/clc-assembly-cell). The whole-genome assemblies of *A. borealis* and *A. sinapina* consisted of 23,459 contigs with a total length of 72,723,723 bp (N50 = 13,708 bp) and 34,632 contigs with a total length of 94,366,584 bp (N50 = 7681 bp), respectively. To find among these contigs those that represent mitochondrial sequences, they were blasted against all mitochondrial basidiomycete sequences available in the NCBI GenBank, and contigs that matched these mitochondrial sequences were selected for further analysis.

The mitogenomes of *A. solidipes* and *A. gallica* were assembled as part of the whole genome sequencing project at the Joint Genome Institute. The complete sequences of their mitogenomes were available as two scaffolds deposited in the JGI Genome portal (http://genome.jgi.doe.gov/Armost1/Armost1.home.html and https://genome.jgi.doe.gov/Armga1/Armga1.home.html, respectively). These scaffolds were retrieved for annotation and comparative analysis in this study.

Mitochondrial contigs found in the total *A. borealis* and *A. sinapina* contig assemblies were verified by mapping these contigs to the *A. solidipes* mitogenome using CLC Genomics Workbench v. 9.0.1 (QIAGEN Bioinformatics, Hilden, Germany; https://www.qiagenbioinformatics.com/products/clc-genomics-workbench). Identified mitochondrial contigs were additionally further confirmed by BLASTn searches against non-redundant nucleotide sequences in the NCBI GenBank database. After identification and confirmation of mitochondrial contigs those of them that displayed overlaps at both ends were used to circularize the mitogenomes using Cyclic DNA Sequence Aligner [68]. Finally, two 116,433 and 103,563 bp single contigs representing the whole mitogenomes of *A. borealis* and *A. sinapina* were arranged and circularized into mitogenomes, respectively. For final verification, raw paired-end sequence reads were mapped to them using CLC Genomics Workbench v 9.0.1.

### RNA isolation, sequencing and mapping

The total RNA was extracted from grown mycelia fixed in RNA*later* (Thermo Fisher Scientific Company, Waltham, Massachusetts, USA) using Qiagen RNeasy Mini Kit (Qiagen, Valencia, CA, USA). The quality and concentration of the RNA were measured using Agilent 2100 Bioanalyzer and Agilent RNA 6000 Nano kit. High quality purified RNA was selected for cDNA library construction. Isolation of mRNA from total RNA was performed using Oligo (dT) magnetic beads. The mRNA treated with fragmentation buffer was used as template for cDNA synthesis. Double-stranded cDNA libraries were constructed using the TruSeq RNA Library Prep Kit v2 (Illumina, San Diego, CA). End-repair, A-tailing, adapter ligation, and library amplification were done for the cDNA library construction followed by cluster generation and sequencing on the Illumina MiSeq platform in the Laboratory of Forest Genomics (Genome Research and Education Center, Siberian Federal University, Krasnoyarsk, Russia) using MiSeq Reagent Kit v2 (2 × 150). Raw sequence data were processed, and adapters were removed. High quality reads were mapped to *A. borealis* and *A. sinapina* mitogenomes using the RNA-Seq module of CLC Genomics Workbench v. 9.0.1 (QIAGEN Bioinformatics, Hilden, Germany; https://www.qiagenbioinformatics.com/products/clc-genomics-workbench).

RNA-seq data for *A. solidipes* and *A. gallica* were downloaded from the NCBI Sequence Read Archive (accession numbers SRR4063418 and SRX5202894, respectively) and used for mapping these two mitogenomes.

### Gene annotation and bioinformatic analyses

Mitogenomes for three *Armillaria* species were checked for homology with other fungal mitogenomes existing in the NCBI GenBank database using the NCBI BLAST algorithm. The mitogenomes were annotated using the MFannot program (http://megasun.bch.umontreal.ca/cgi-bin/mfannot/mfannotInterface.pl) with default settings. Multiple ORFs were analyzed by a BLASTx homology search against protein database in the NCBI GenBank database. Intron-exon boundaries were verified using RNAweasel (http://megasun.bch.umontreal.ca/RNAweasel). Large (*rnl*) and small (*rns*) subunits of the mitochondrial ribosome genes were predicted using both BLASTn and RNAweasel. The tRNA genes were discovered using ARAGORN [69] and tRNAscan-SE [70] tools. Duplicated mtDNA sequences were identified by local BLASTn searches of mtDNAs against themselves with the e-value cut-off of 10^− 5^. To identify sequence repeats in intergenic regions of the mitogenomes SciRoKo [71] and Tandem Repeat Finder [72] were used with defaults parameters. Whole mitogenome alignments to identify syntenic blocks in mitogenomes of *A. borealis*, *A. solidipes*, *A. sinapina* and *A. gallica* were performed using MAUVE 2.3.1 [73]. We also determined the frequency of codon usage in 14 mitochondrial genes using the web-based Sequence Manipulation Suite (http://www.bioinformatics.org/sms2/codon_usage.html) with the fungal mitochondrial genetic code 4.

### Phylogeny

Phylogenetic analysis was performed using protein sequences representing totally 3645 amino acids of 14 concatenated protein-coding mitochondrial genes (*nad1, nad2, nad3, nad4, nad4L, nad5, nad6, cob, cox1, cox2, cox3, atp6, atp8, atp9*) partitioned from mitogenomes of four *Armillaria* species described in this study and 21 fungal taxa downloaded from GenBank (their names and accession numbers are provided in Additional file 6: Table S6. The maximum likelihood (ML) method in the IQTree v. 1.5.6 software was used to generate a phylogenetic tree [74]. Sequence alignments were produced using the MAFFT algorithm implemented in SeaView v. 4.7 ([75]; http://doua.prabi.fr/software/seaview). Poorly aligned regions were trimmed using GBlocks [76]. The best models of evolution for amino acid sequences were selected by PartitionFinder2 v. 2.1.1 [77]. LG + G + F were determined as the best models for the *atp6, atp8, cox3, nad2, nad3, nad4, nad5*, and *nad6* genes and LG + I + G + F models for the *atp9, cob*, *cox1, cox2, nad1,* and *nad4L* genes, where LG – one of general amino-acid replacement models introduced by [78], +I – invariable site model (one of the common rate heterogeneity across sites models allowing for a proportion of invariable sites) [79], *+G* - discrete Gamma model [80], and *+ F* - empirical base frequencies. To determine the statistical support of the recovered nodes in the ML phylogenetic tree, the ultrafast bootstrap approximation was performed with 10,000 replicates.

## Additional files

Additional file 1: **Table S1.** Tandem repeats detected in four *Armillaria* mitogenomes using Tandem Repeats Finder. (XLSX 14 kb)

Additional file 2: **Table S2.** Microsatellite or simples equence repeat (SSR) loci in four *Armillaria* mitogenomes. (XLSX 11 kb)

Additional file 3: **Table S3.** Comparison of codon usage of 14 oxidative phosphorylation genes **and** the codon-anticodon recognition pattern of tRNA genes identified in *A. borealis, A. gallica, A. sinapina*, and *A. solidipes.* The number of plus signs indicates the presence and numbers of the respective tRNA gene. (XLSX 17 kb)

Additional file 4: **Table S4.** Classification of introns in mitogenomes of four *Armillaria* species. (XLSX 9 kb)

Additional file 5: **Table S5.** Mapping *Armillaria* transcriptome reads on mitogenomes. (DOCX 14 kb)

Additional file 6: **Table S6.** Names and accession numbers for 21 *Basidiomycota* species downloaded from GenBank and used for phylogenetic analysis. (XLSX 9 kb)

## Abbreviations

*atp6* and *atp8*: Genes for ATP synthase subunits 6 and 8
*bp*: Base pair
*cob*: Gene for cytochrome b
*cox1, cox2* and *cox3*: Genes for cytochrome c oxidase subunits 1, 2 and 3;
*kb*: Kilo base pair
*Mt*: Mitochondrial
*nad1, nad2, nad3, nad4, nad4L, nad5* and *nad6*: Mitochondrial genes for NADH dehydrogenase subunits 1–6 and 4 L
*ORF*: open reading frame
*rnS* and *rnL*: Genes for small and large subunits of ribosomal RNA
*rRNA*: Ribosomal RNA
*trnA* or *A*: tRNA gene for alanine
*tRNA*: Transfer RNA
*trnC* or *C*: tRNA gene for cysteine
*trnD* or *D*: tRNA gene for aspartic acid
*trnE* or *E*: tRNA gene for glutamic acid
*trnF* or *F*: tRNA gene for phenylalanine
*trnG* or *G*: tRNA gene for glycine
*trnH* or *H*: tRNA gene for histidine
*trnI* or *I*: tRNA gene for isoleucine
*trnK* or *K*: tRNA gene for lysine
*trnL*_*1*_ or *L*_*1*_: tRNA gene for leucine (anticodon NAG)
*trnL*_*2*_ or *L*_*2*_: tRNA gene for leucine (anticodon YAA)
*trnM* or *M*: tRNA gene for methionine
*trnN* or *N*: tRNA gene for asparagine
*trnP or P*: tRNA gene for proline
*trnQ* or *Q*: tRNA gene for glutamine
*trnR* or *R*: tRNA gene for arginine
*trnS*_*2*_ or *S*_*2*_: tRNA gene for serine (anticodon NGA)
*trnS*_*1*_ or *S*_*1*_: tRNA gene for serine (anticodon NCU)
*trnT* or *T*: tRNA gene for threonine
*trnV* or *V*: tRNA gene for valine
*trnW* or *W*: tRNA gene for tryptophan
*trnY* or *Y*: tRNA gene for tyrosine
μl: Microliter
