# Supplementary material for: Mobile genetic elements explain size variation in the mitochondrial genomes of four closely-related Armillaria species
Source: BMC Genomics. 2019 May 8;20:351. doi: 10.1186/s12864-019-5732-z (PMC6506933; doi:10.1186/s12864-019-5732-z)
Supplement: Supplementary file 5 — Table S5. Mapping Armillaria transcriptome reads on mitogenomes. (DOCX 14 kb) [file 12864_2019_5732_MOESM5_ESM.docx]

**Table S5** Mapping *Armillaria* transcriptome reads to their mitogenomes

| Species | | Transcriptome reads | | Mitochondrial genes covered by reads (%) |
| --- | --- | --- | --- | --- |
| transcriptome | mitogenome | total | mapped (%) |  |
| *A. borealis*^1^ | *A. borealis* | 2,371,666 | 258,471 (10.90) | 17 (100) |
| *A. sinapina*^1^ | *A. sinapina* | 1,844,578 | 227,565 (12.34) | 16 (94) |
| *A. gallica*^2^ | *A. gallica* | 6,000,000 | 566 (0.01)^3^ | 11 (65) |
| *A. solidipes*^2^ | *A. solidipes* | 6,000,000 | 442 (0.01)^3^ | 8 (47) |
| *A. borealis*^1^ | *A. gallica* | 2,371,666 | 251,265 (10.59) | 16 (94) |
| *A. borealis*^1^ | *A. solidipes* | 2,371,666 | 255,744(10.78 ) | 16 (94) |

^1^ Authors‘ transcriptome data

^2^ Transcriptome data obtained from NCBI Sequence Read Archive (accessions SRR4063418 and SRX5202894)

^3^ Low mapping coverage can be likely explained by a very low quality of transcriptome reads for *A. gallica* and *A. solidipes*, long stretches of which contained anonymous nucleotides (N)
